# Radiomic features based on Hessian index for prediction of prognosis in head-and-neck cancer patients

**DOI:** 10.1038/s41598-020-78338-7

**Published:** 2020-12-04

**Authors:** Quoc Cuong Le, Hidetaka Arimura, Kenta Ninomiya, Yutaro Kabata

**Affiliations:** 1 Department of Health Sciences, Graduate School of Medical Sciences, Kyushu University, Fukuoka, Japan; 2 Department of Health Sciences, Faculty of Medical Sciences, Kyushu University, Fukuoka, Japan; 3 Institute of Mathematics for Industry, Kyushu University, Fukuoka, Japan

**Keywords:** Oncology, Risk factors, Cancer, Head and neck cancer

## Abstract

This study demonstrated the usefulness of radiomic features based on the Hessian index of differential topology for the prediction of prognosis prior to treatment in head-and-neck (HN) cancer patients. The Hessian index, which can indicate tumor heterogeneity with convex, concave, and other points (saddle points), was calculated as the number of negative eigenvalues of the Hessian matrix at each voxel on computed tomography (CT) images. Three types of signatures were constructed in a training cohort (n = 126), one type each from CT conventional features, Hessian index features, and combined features from the conventional and index feature sets. The prognostic value of the signatures were evaluated using statistically significant difference (*p* value, log-rank test) to compare the survival curves of low- and high-risk groups. In a test cohort (n = 68), the *p* values of the models built with conventional, index, combined features, and clinical variables were 2.95 $\times$ 10^–2^, 1.85 $\times$ 10^–2^, 3.17 $\times$ 10^–2^, and 1.87 $\times$ 10^–3^, respectively. When the features were integrated with clinical variables, the *p* values of conventional, index, and combined features were 3.53 $\times$ 10^–3^, 1.28 $\times$ 10^–3^, and 1.45 $\times$ 10^–3^, respectively. This result indicates that index features could provide more prognostic information than conventional features and further increase the prognostic value of clinical variables in HN cancer patients.

## Introduction

Head-and-neck (HN) cancer is the sixth leading cancer worldwide and more than 90% of HN cancers are squamous cell carcinoma^1^ (HNSCC). Each year, globally, more than 500,000 HNSCC patients are newly diagnosed, of which 40,000 are in the United States of America (USA). Around 8000 deaths are reported in the USA annually^2,3^. The loco-regional control rate in HN cancer is approximately 90%, but the 5-year overall survival probability is only about 50%^4^, mainly due to distant metastasis and second primary cancer^5,6^. Despite recent developments in cancer treatment, individual patients with HNSCC still exhibit different outcomes^7^. For oropharyngeal cancer, which is the dominant population of patients in this study, Ang et al.^8^ reported that human papillomavirus (HPV) could be a strong and independent prognostic factor (3-year survival rate: 82.4% for HPV positive and 57.1% for HPV negative patients). Therefore, the prediction of prognosis prior to the treatment of HN cancer could be critical for the selection of more personalized treatment approaches for each patient.

Radiomics, which is a quantitative approach in image analysis, could possess prognostic power and potentially be used to evaluate tumor heterogeneity and treatment outcome. Radiomic features quantifying tumor intensity and texture are often extracted from medical images such as computed tomography (CT)^9^ and positron emission tomography (PET) images^10–12^. The radiomics approach proved that tumors with greater heterogeneity on CT value spatial distributions in HN cancer patients have a poorer prognosis^13^. Parmar et al.^14^ proposed an approach for the classification of patients into shorter than, and longer than three years survivors, by using thirteen feature selection algorithms and eleven classification methods with conventional radiomic signatures. However, so far, there have been no reports of the area under the receiver operating characteristic curve (AUC) exceeding 0.8. Ou et al.^15^ investigated the prognostic powers of radiomic signatures with conventional texture features obtained from CT images in locally advanced HN cancers patients, for predicting the 5-year overall survival after concurrent chemoradiotherapy (CRT) or bioradiotherapy (BRT). In their study, a radiomic signature alone was reported to achieve an AUC of 0.67. When an HPV/p16 status was combined with the radiomic signatures, the reported AUC was 0.78. These results motivated us to develop new radiomic features for improving the performance of the prognosis prediction, because the conventional texture features may have reached a plateau.

Radiomic features could have the potential to quantify tumor heterogeneity which can be associated with patients’ prognosis^16–18^, tissue classification^19^, and tumor recurrence^20,21^. The spatially local heterogeneity of physical values (e.g., CT values) could be categorized into local bright points (convex points), dark points (concave points), or others (saddle points). We assumed that these physical value distributions with local heterogeneity can be represented by the Hessian index of differential topology, which is calculated as the number of negative eigenvalues of the Hessian matrix^22^. Because the Hessian indices are capable of indicating a convex point with an index of 3, a hyperbolic paraboloid (saddle) point with the indices of 1 or 2, and a concave point with an index of 0, they could be employed to reflect the distribution of physical values on a CT image. The Hessian index is invariant under changing of coordinate systems^22^.

The difference between past studies and ours was that we calculated the Hessian index of each voxel to obtain index images (i.e. a map of Hessian indices) from which radiomic features were then extracted. On the other hand, Geneshan et al.^23^ and Chaddad et al.^24^ employed only the Laplacian-of-Gaussian filter, and Parekh et al.^25^ and Prasanna et al.^26^ made conventional feature maps and new radiomic features, respectively. These ideas were different from the Hessian index features. Furthermore, Pyradiomics^27^, which is a useful open-source software in radiomics analysis, does not include the calculation functions of the Hessian matrices and index features.

We have developed a radiomic approach based on the Hessian index for the prediction of patients’ prognosis in HN cancer patients. The Hessian indices at each voxel on a CT image were calculated to generate index images from which index features were extracted. We hypothesized that index features could provide more prognostic information than conventional features in characterizing tumor heterogeneity related to the patients’ prognosis.

## Results

### Construction of radiomic signatures

**Figure 1:**
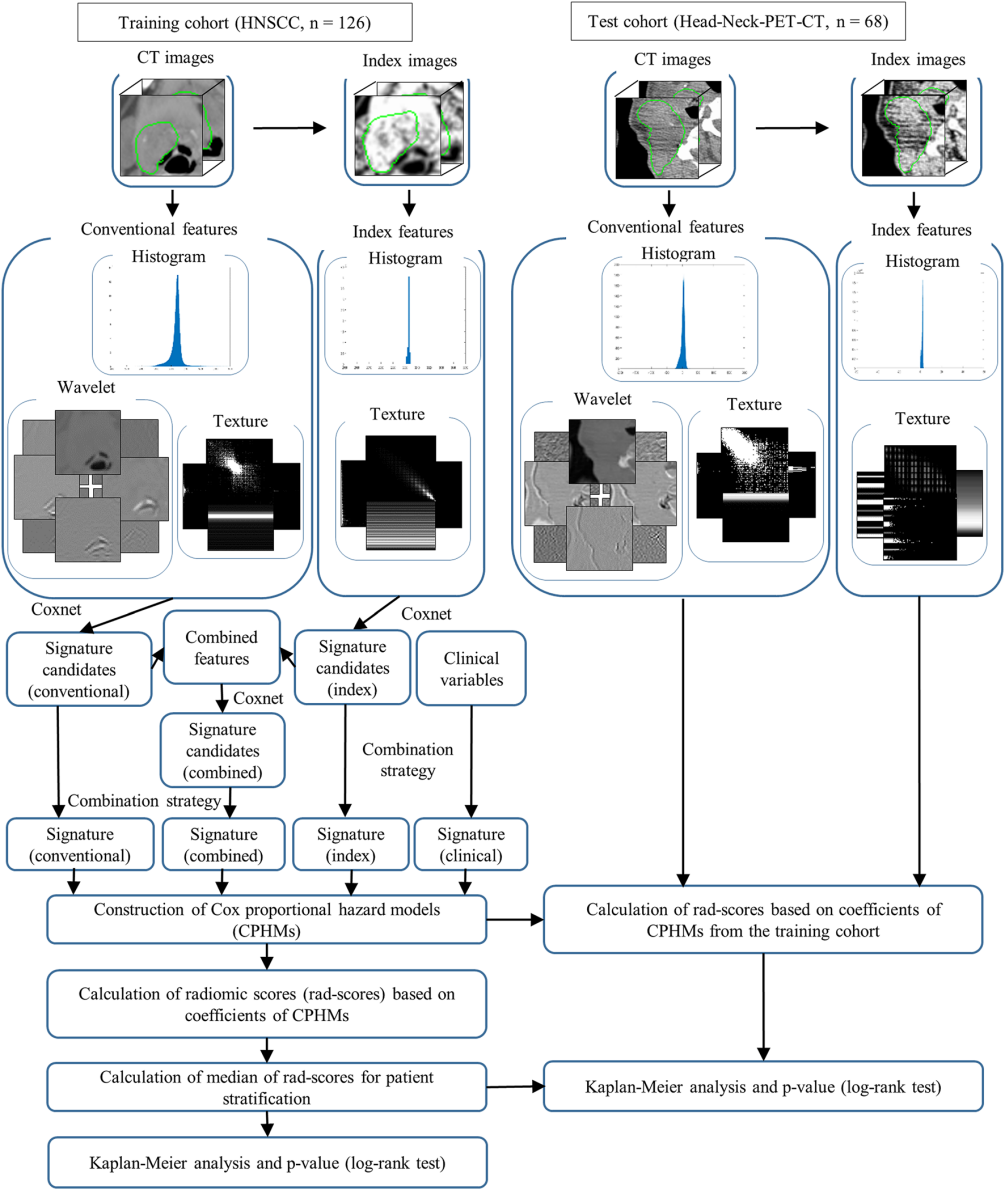
An overall workflow used in this study. First, index images were derived from CT images. Then, conventional and index features were calculated from CT and index images, respectively, by applying calculation methods based on histogram and texture features to both images. Wavelet features, which are based on wavelet decomposition filters, were only extracted from CT images. Wavelet decomposition filters were not applied to the index images because they might introduce rotational dependency to the index images which are considered invariant under changing of coordination systems. Next, a Coxnet algorithm was employed to select signature candidates for conventional, index, and combined features. All clinical variables were considered signature candidates. A combination strategy was then applied to construct multiple signatures containing from 1 to 12 features from conventional, index, combined, and clinical-variable signature candidates. CPHMs were then trained using the constructed signatures and clinical variables, and radiomic scores (rad-scores) were computed based on model’s coefficients. The patients were stratified into low- and high-risk groups based on the medians of the rad-scores. Finally, a Kaplan–Meier analysis with *p* value from the log-rank test were employed to evaluate the models’ performance. The model yielded the lowest *p* value for a test cohort was considered the best model, and the corresponding feature set was considered to have the highest prognostic power.

Radiomic signatures were constructed from signature candidates, which are features having the relationship with patients’ prognosis, using a combination strategy with Cox proportional hazard model^28^ (CPHM). This combination strategy was inspired by the works of Vallières et al.^29^ and is detailed in Supplementary Subsection 3.1. In this study, a Coxnet algorithm^30,31^ with optimized blending parameter $\alpha$ was applied to the conventional and index feature sets for selection of signature candidates. Signature candidates from the conventional and index feature sets were mixed to form a combined feature set, to which the Coxnet algorithm were then applied to select signature candidates for the combined feature set (Fig. 1). Moreover, clinical variables, i.e. age, T stage, N stage, TNM stage, tumor volume, and HPV status, were integrated with radiomic features to investigate any potential increments in term of prediction of prognosis.

For conventional, index, and combined feature sets, the optimal values of $\alpha$ for the Coxnet algorithm were 0.1, 0.08, and 0.01, respectively. The Coxnet algorithm was applied to the three feature sets using the corresponding optimal values of $\alpha$ for the selection of signature candidates. As a result, 88, 65, and 153 signature candidates were selected from the conventional, index, and combined feature sets, respectively. All the six clinical variables were considered signature candidates.

For a given feature set, twelve signatures consisting of 1 to 12 features were constructed. We only constructed signatures containing up to twelve features, because the number of features in the signature should not be larger than one tenth of the number of patients in the training cohort^32^ (n = 126). For clinical variables, five signatures constituting 1 to 5 clinical variables were constructed. It should be notice that HPV status was not used in this multivariate analysis because this information was not entirely available in our datasets (training cohort: 31/126, test cohort: 17/68).

### Building predictive models using radiomic signatures

For each feature set, twelve signatures constituting 1–12 features were constructed based on the combination strategy. A CPHM was then built in the training cohort for predicting patients’ prognosis. The prognostic power of the CPHMs was evaluated based on statistically significant difference (*p* value, log-rank test) between two Kaplan–Meier survival curves that was stratified using the median of the radiomic scores (rad-scores). A rad-score is calculated for each patient as a linear combination between each feature in the signature weighted by the corresponding coefficient from the CPHM. The coefficients and medians obtained from the training cohort were applied to the test cohort for calculation of rad-scores and stratification of patients, respectively. A log-rank *p* value was then calculated for test cohort. For a given feature set, the model yielding the lowest *p* value in the test cohort was consider the best model, and the corresponding signature was considered the best signature.

Furthermore, the impact of streak artifact on the best model was investigated based on an artifact group (n = 20) and a non-artifact group (n = 48) in the test cohort (n = 68). The test cohort was divided by a medical physicist (H.A.) into artifact and non-artifact groups by observing CT images of each patient slice by slice. If a patient had no slices containing artifact within tumor regions, that patient was assigned to the non-artifact group, otherwise the artifact group. For each feature set, the above-mentioned Kaplan–Meier procedure was employed to obtain the log-rank *p* values in the artifact and non-artifact groups. The difference between the *p* values in the two groups was then calculated. The feature set yielded the lowest difference in *p* value was considered least affected by streak artifact.

### Building predictive models using clinical variables

For clinical variables (e.g. age, T stage, N stage, TNM stage, and tumor volume), five signatures constituting 1–5 variables were constructed based on the combination strategy. A CPHM was built for each clinical-variable signature and the above-mentioned Kaplan–Meier procedure was employed for selecting the best signature. HPV status was analyzed separately in a univariate analysis because this information was not fully available in our analyzed cohorts (training cohort: 31/126, test cohort: 13/68). Moreover, tumor volume was also analyzed separately in a univariate analysis because this variables was reported as an independent prognostic factor in HN cancer^33^.

### Building predictive models using radiomic signatures and clinical variables

For each feature set, the radiomic signatures were integrated with clinical-variable signatures to investigate any potential increment in prognostic power, compared to clinical variables alone. Sixty integrated signatures (12 radiomic signatures $\times$ 5 clinical-variable signatures) was constructed and a CPHM was built for each integrated signature. The best integrated signature was identified using the above-mentioned Kaplan–Meier procedure.

### Evaluation of prognostic power

**Figure 2:**
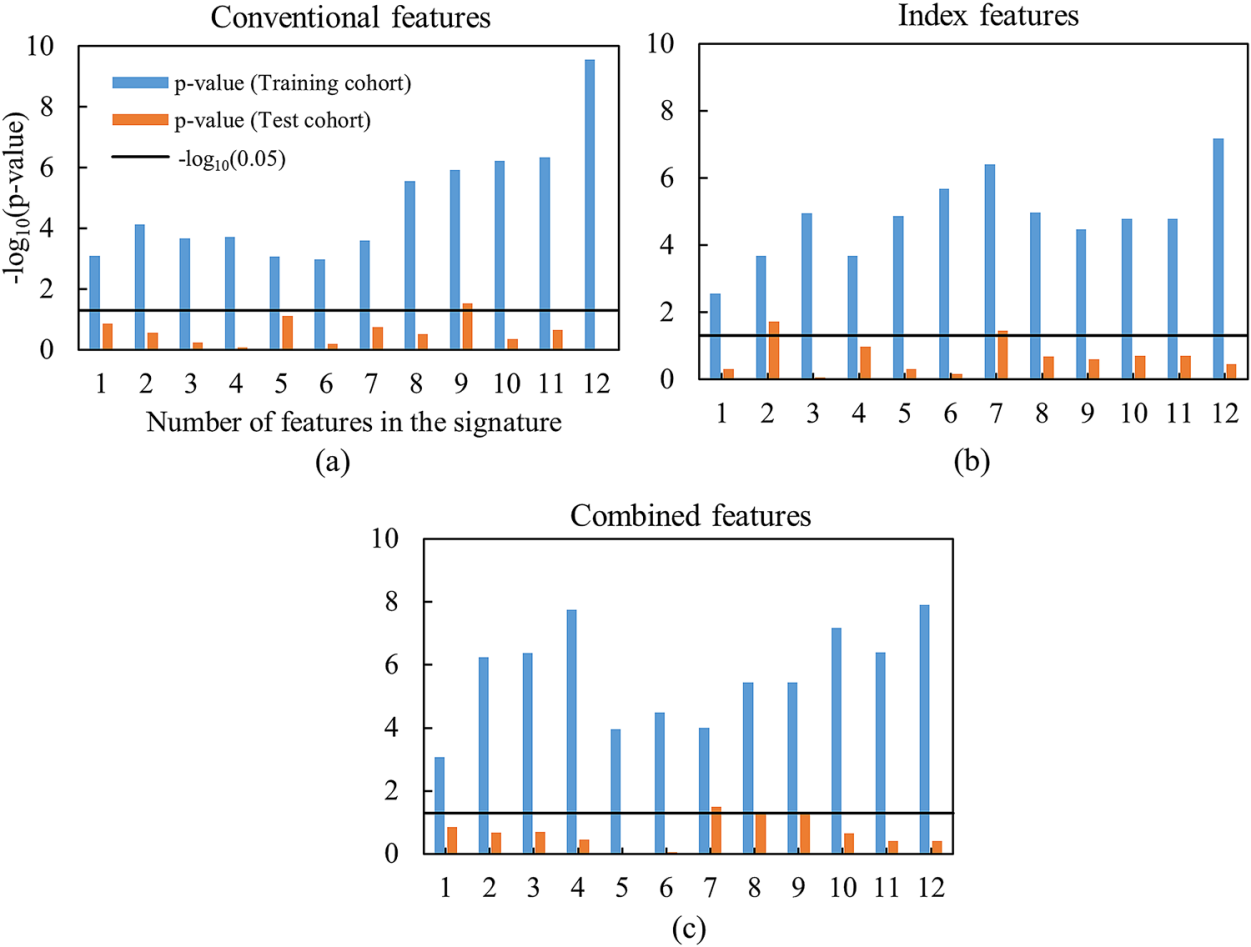
Performance of the CPHMs built in the training and test cohorts using (**a**) conventional, (**b**) index, and (**c**) combined feature sets.

For a given feature set, twelve signatures and corresponding CPHMs were built. The model yielded the lowest *p* value in the test cohort was considered the best model, and the corresponding signature was considered the best signature. Figure 2 exhibits the *p* values from the log-rank test of the twelve CPHMs in the training and test cohorts from the three feature sets. According to Fig. 2, the best signatures in the conventional, index, and combined feature sets consisted of 9, 2, and 7 features, respectively. The name of features in the signature for each feature set is detailed in Supplementary Table S2. The corresponding log-rank *p* values of the best models in the training and test cohorts were 1.17 $\times$ 10^–6^ and 2.95 $\times$ 10^–2^ for conventional feature set, 2.10 $\times$ 10^–4^ and 1.85 $\times$ 10^–2^ for index feature set, and 10^–4^ and 3.17 $\times$ 10^–2^ for combined feature set, respectively. It can be seen that among three best models, the model built using index features possessed the highest performance in the test cohort.

The impact of streak artifact on the best models was investigated by dividing the test cohort (n = 68) into an artifact group (n = 20) and a non-artifact group (n = 48). The best models from each feature set were evaluated in the artifact and non-artifact group. The log-rank *p* values of the best models in the artifact and non-artifact groups, and the difference in *p* value between the two groups were 5.32 $\times$ 10^–1^, 1.07 $\times$ 10^–2^, and 5.21 $\times$ 10^–1^ for conventional features; 6.58 $\times$ 10^–2^, 5.05 $\times$ 10^–2^, and 1.53 $\times$ 10^–2^ for index features; and 2.17 $\times$ 10^–1^, 7.55 $\times$ 10^–2^, and 1.42 $\times$ 10^–1^ for combined features, respectively.

**Figure 3:**
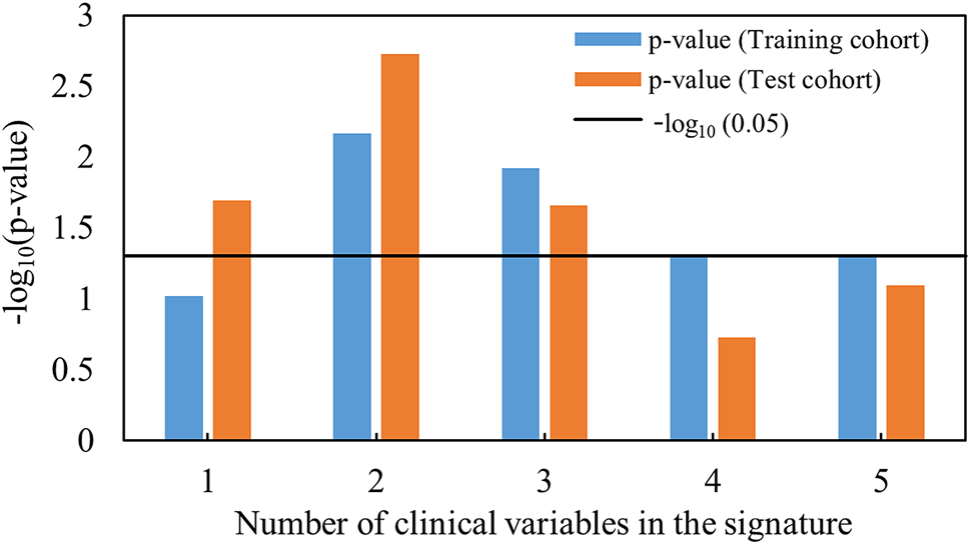
Performance of models built using clinical variables only in the training and test cohorts.

In a multivariate analysis involving clinical variables (e.g. age, T stage, N stage, TNM stage, and tumor volume), five signatures were constructed and five corresponding CPHMs were built. Figure 3 exhibits Kaplan–Meier *p* values of the five models in the training and test cohorts. By examining Fig. 3, the model built with two clinical variables (i.e. T stage and TNM stage) yielded the highest performance with the corresponding *p* values in the training and test cohorts were 6.80 $\times$ 10^–3^ and 1.87 $\times$ 10^–3^, respectively.

**Figure 4:**
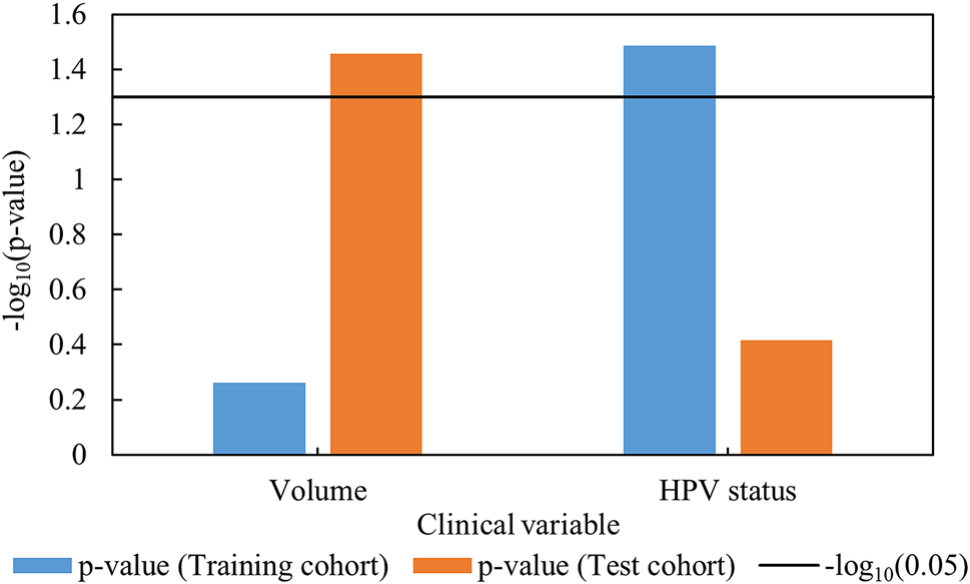
Performance of the CPHMs built using tumor volume and HPV status in a univariate analysis.

Figure 4 exhibits the performance of two CPHMs built using solely tumor volume and HPV status in a univariate analysis. According to Fig. 4, the CPHM built solely with tumor volume yield the log-rank *p* values of 1.54 $\times$ 10^–3^ and 1.62 $\times$ 10^–1^ in the training and test cohorts, respectively. The model built with HPV status alone yielded the log-rank *p* values of 3.27 $\times$ 10^–2^ and 3.83 $\times$ 10^–1^ in the training and test cohorts, respectively. It should be noticed that the model using HPV status were built with only 31 of 126 patients in the training cohort and evaluated using 17 of 68 patients in the test cohort because this information was not fully available in our analyzed cohorts.

**Figure 5:**
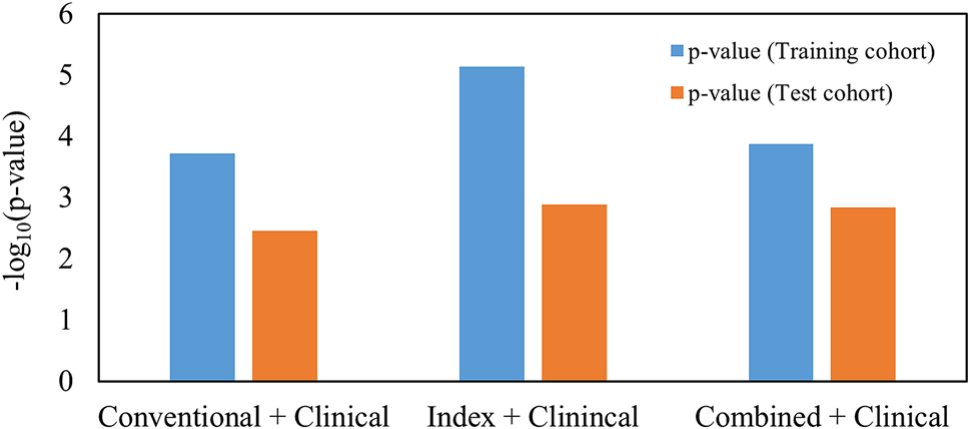
Performance of the best model built using the combination between clinical variables and conventional, index, and combined features.

When radiomic signatures were integrated with clinical-variable signatures, the best integrated signature in the conventional, index, and combined feature set constituted 5 features and 5 clinical variables, 9 features and 5 clinical variables, and 7 features and 5 clinical variables, respectively. The names of features and clinical variables in the integrated signatures from each feature set are detailed in Supplementary Table S2. Figure 5 shows the Kaplan–Meier *p* values of the best models built using the signature integrated between clinical variables and conventional, index, and combined features. By examining Fig. 5, it can be seen that the model built using index features and clinical variables possessed the highest prognostic power with Kaplan–Meier *p* value in the training and test cohorts of 7.33 $\times$ 10^–6^ and 1.28 $\times$ 10^–3^, respectively.

## Discussion

Radiomic studies were reported to have the association with patients’ prognosis^16–18^, tissue classification^19^, and tumor recurrence^20,21^. This study described the usefulness of radiomic features based on the Hessian index. By using CPHMs and the Kaplan–Meier analysis, the index features exhibited potential to stratify patients into low- and high-risk group, compared to conventional and combined features. We believe that the index features in this work could be generalized to other type of medical images and further enhance their diagnostic value.

Two different publicly available dataset from the TCIA database^34^, i.e. HNSCC^35^ and Head-Neck-PET-CT^36^, were used as training and test cohorts, respectively. Using two independent cohorts allowed us to perform a completely external validation of our predictive models. This type of external validation enables us to investigate the robustness of our models more correctly, compare to internal validation or random splitting of the dataset^37^.

The training and test cohorts included various CT images which were acquired by different institutions, manufacturers, scanner models, pixel sizes, X-ray tube voltages, exposures (mAs), pitches, and reconstruction filters. Conventional radiomic (texture) features extracted from CT images may reportedly depend on CT image acquisition parameters^38^. On the contrary, the model built using index features achieved higher performances, compared to conventional and combined features. Consequently, the log-rank *p* values in the training and test cohort were 1.17 $\times$ 10^–6^ and 2.95 $\times$ 10^–2^ for conventional features, 2.10 $\times$ 10^–4^ and 1.85 $\times$ 10^–2^ for index features, and 10^–4^ and 3.17 $\times$ 10^–2^ for combined features, respectively. This result suggests CPHMs built using index features could be more robust to variation in CT acquisition techniques.

Cancer stage and treatment method are known factors that could affect survival. In this study, we used a training cohort in which the patients were at various stages and were treated with multiple treatment methods in order to investigate the impact of those confounding factors on the final models’ performance. The models were evaluated in a test cohort which was more homogeneous in term of cancer stage and treatment methods. As a result, the model built using index features (Fig. 2b) outperformed models using conventional (Fig. 2a) and combined features (Fig. 2c). The log-rank *p* values in the training and test cohort were 1.17 $\times$ 10^–6^ and 2.95 $\times$ 10^–2^ for conventional features, 2.10 $\times$ 10^–4^ and 1.85 $\times$ 10^–2^ for index features, and 10^–4^ and 3.17 $\times$ 10^–2^ for combined features, respectively. This result suggests that the model built using index features could be used more effectively in a dataset with heterogeneous treatment methods and cancer stage, compared to conventional and combined features.

Image noise, which could affect the calculation of second derivative^39,40^, might also affect index features. By applying three Gaussian filters to the CT images, image noise could be reduced and hence its effects on the index images could be alleviated. Additionally, two averaging filters were then convolved with the index images to reduce index noise induced by any possible remaining noise from CT images. By using these techniques, the impact of image noise on index features could be minimized.

Signature candidates associated with the survival time were selected by using the Coxnet algorithm. Parmar et al.^14^ examined thirteen filter-based feature selection algorithms using the binary classification of patients based on the survival time. However, these methods, which excluded censored patients from the dataset, could possibly reduce the generalizability of the signature candidates. In this study, since the Coxnet algorithm is based on the multivariate Cox proportional hazard regression model that treats the survival time as a continuous variable and accounts for patient censorship information, all patients could be enrolled for selecting signature candidates. Consequently, signature candidates selected by the Coxnet could have high generalizability and correlation to survival time. Moreover, the CPHMs, which uses both time-to-event and censoring data for patient stratification, could take into account the high generalizability of the signature candidates produced by the Coxnet. Using the Coxnet algorithm and CPHM gives us a consistency between feature selection and model building, and could further help us to prevent any potential of information loss.

Clinical variables (i.e. age, T stage, N stage, TNM stage, tumor volume, and HPV status) were compared with the conventional, index, and combined features in term of prediction of prognosis. As a result, a combination between T stage and TNM stage (Fig. 3) produced a model with highest performance whose *p* value in the training and test cohorts were 6.80 $\times$ 10^–3^ and 1.87 $\times$ 10^–3^, respectively. Although HPV status was reported to be an independent predictor in HN cancer^8,41^, the CPHMs built using conventional, index, and combined features (Fig. 2) outperformed the model built solely with HPV status (Fig. 4) in this study. We believe that because of a scarce number of patients with HPV status used for training (n = 31/126), the model built using HPV status alone could not possess high prognostic power, compared to the radiomic models. Moreover, the CPHMs built using conventional, index, and combined features (Fig. 2) also outperformed the model built using tumor volume alone (Fig. 4). This result agrees with past studies^42,43^. Although tumor volume is an independent prognostic factor in HN cancer^33^, Hatt et al.^42^ reported that texture analysis may provide more valuable information compared to tumor volume alone when the tumor is larger than 10 cm^3^. In this study, because the majority patients have tumors exceeding 10 cm^3^ (training cohort: 108/126, test cohort: 51/68), radiomic features were found to obtained more prognostic power, compared to tumor volume. Furthermore, EBV status was reported to be as useful biomarker in prediction of prognosis for nasopharyngeal cancer patients^44,45^. We could not performed this task because EBV status was not available in our dataset.

Conventional, index, and combined signatures were also integrated with clinical-variable signatures to investigate any potential increment in prognostic power, compared to when clinical-variable signatures were using alone (Fig. 3, training cohort: *p* value  = 6.80 $\times$ 10^–3^, test cohort: *p* value  = 1.87 $\times$ 10^–3^, log-rank test). Index features added more prognostic value to clinical data, compared to combined features. No increment in prognostic value was observed for conventional features (Fig. 5). The log-rank *p* values in the training and test cohorts were 1.90 $\times$ 10^–4^ and 3.53 $\times$ 10^–3^ for conventional features, 7.33 $\times$ 10^–6^ and 1.28 $\times$ 10^–3^ for index features, and 1.32 $\times$ 10^–4^ and 1.45 $\times$ 10^–3^ for combined features. This result suggests that index features could add more prognostic value to clinical data. Ou et al.^15^ reported that a combination between HPV/p16 status and radiomic signature could further increase the model performance. We could not perform this task in our study because HPV status was not fully available (training cohort: 31/126, test cohort: 13/68) in our dataset.

This study has four limitations. Firstly, the number of selected patients in this study was relatively small, which may reduce the prognostic power of predictive models. More patients will be included in future studies. Secondly, signatures constructed using a dataset with homogeneous treatment method and cancer stage could be different from ours. We included patients with various treatment methods and cancer stage to investigate the effect of those confounding factors on the models’ performance. Although the evaluation using a test cohort suggested that our radiomic models could be effective to be used in a dataset with multiple treatment methods and cancer stages, the prediction performance may vary if a dataset with one specific treatment method and cancer stage was used for model building. Thirdly, this study did not take into account the variations of CT imaging protocols. Since inter-scanner variability was reported to affect conventional features^46,47^, future studies are needed to examine the impact of this variability on index features. Finally, head-and-neck radiomics are sensitive to noise and artifact. Wei et al.^48^ reported that streak artifacts could decrease the performance of HN radiomics models. Methods to remove streak artifacts prior to the generation of index images, such as the one proposed by Wei et al.^48^, will be employed in our future studies.

## Methods

### Clinical datasets availability

This study was conducted using two publicly available dataset from the works of Grossberg et al.^35^ and Vallières et al.^36^ as a training and an independent test cohort, respectively. The datasets used in this study are made publicly available by Grossberg et al.^35^ and Vallières et al.^36^ on The Cancer Imaging Archive (TCIA) database^34^ at: http://www.cancerimagingarchive.net. Table 1 details the patients’ characteristics in the training and test cohorts in this study. The training cohort constitutes 126 cancer patients with pathologically proven HNSCC^35^. Five nasopharyngeal cancer patients have non-keratinizing subtype (World Health Organization type 2) and their Epstein-Barr virus (EBV) status was not available. The patients were treated with four treatment methods, i.e. concurrent chemoradiotherapy, chemotherapy, surgery, or external beam radiotherapy. The CT images of 89 patients were excluded from the original dataset because their metadata could not be entirely imported using the function for reading Digital Imaging and Communications in Medicine (DICOM) files (*dicominfo* in Matlab 2018a). The test cohort constitutes sixty eight patients who were randomly selected from the publicly available dataset Head-Neck-PET-CT^36^, which is different from the training cohort. Neither subtype information nor EBV status was available for eleven nasopharyngeal cancer patients in the test cohort. The patients were treated using radiotherapy or chemoradiotherapy. The staging of patients in the training and test cohorts was performed according to the American Joint Committee on Cancer 7th edition.

**Table 1:** Patients’ characteristics in training and test cohorts.

	Training cohort (n = 126)	Test cohort (n = 68)
Age (mean)	29–91 (56.79)	18–84 (64.63)
Gender (male/female)	107/19	54/14
Overall stage (I/II/III/IV/IVA/IVB)	3/2/15/0/95/11	0/7/12/7/36/6
Event/censor	81/45	45/23
Site (larynx/oropharynx/nasopharynx/hypopharynx)	0/113/5/8	11/38/11/8

In the training cohort, eighteen patients (n = 14%) were treated using concurrent chemoradiotherapy, chemotherapy, and surgery. Fifty four patients (n = 44%) were treated using concurrent chemoradiotherapy and chemotherapy. Eighteen patients (n = 14%) were treated using external beam radiotherapy and surgery, and thirty six patients (n = 28%) were treated using external beam radiotherapy alone. CT images were acquired using two CT scanners of GE Healthcare (LightSpeed and Discovery CT750HD) and three CT scanners of Philips (Mx8000 IDT, Brilliance 64, and PQ5000) with 512 $\times$ 512 pixels, in-plane pixel sizes of 0.64–0.98 mm, slice thicknesses of 2.50–3.75 mm, and 90–348 slices (mean 149.06). The CT images were produced at X-ray tube voltages from 100 to 130 kV (median 120 kV) and exposures from 51 to 328 mAs (median 283 mAs). Pitch information (from 0.891 to 0.942, median 0.938) was only available for eighty-three patients, and CT images of forty-one patients were contrast-enhanced. Low-pass filters using a Philips Healthcare’s B kernel and GE Healthcare’s standard kernel were used to reconstruct CT images for ninety-three patients while reconstruction method for the other thirty-three patients was unavailable. Information about adaptive statistical iteration reconstruction (ASIR) was unavailable for all patients. Anisotropic CT images and gross tumor volume (GTV) regions were transformed into isotropic images with an isovoxel size of 0.98 mm, using cubic and shape-based interpolation^49^. GTV contours of the patients were delineated using a Pinnacle image server (version 9, Philips Radiation Oncology Systems, Fitchburg, WI) in accordance with the International Commission on Radiation Units and Measurements Reports 50 and 62 by expert radiation oncologists with 5 years or post-residency experience at The University of Texas MD Anderson Cancer Center. The contours were then checked at a quality assurance conference in the HN radiation oncology section^35^. The effective diameter of GTVs has the range of 12–74 mm (mean 40 mm).

In the test cohort, fifty eight patients (n = 85%) were treated with chemoradiotherapy while the other ten patients (n = 15%) were treated with radiotherapy. CT images were acquired using three hybrid PET/CT scanners manufactured by GE Healthcare (Discovery ST and Discovery STE) and one manufactured by Philips (GeminiGXL 16) with 512 $\times$ 512 pixels, in-plane pixel sizes of 0.98–1.37 mm, and slice thicknesses of 1.50–3.75 mm. The CT images were acquired at X-ray tube voltages from 100 to 140 kV (median 140 kV) and exposures from 29 to 394 mAs (median 70 mAs). A pitch value of 1.75 was available for only nineteen patients and CT images of forty-six patients were contrast-enhanced. For all patients, low-pass filters using Philips Healthcare’s B and C kernels, and GE Healthcare’s standard and soft kernels were used to reconstruct CT images and information about ASIR technique was unavailable. Anisotropic CT images and gross tumor volume (GTV) regions were transformed into isotropic images with an isovoxel size of 0.98 mm, using cubic and shape-based interpolation^49^. GTV contours were delineated by expert radiation oncologists from four institutions in Canada (i.e. Hôpital général juif, Centre hospitalier universitaire de Sherbooke, Hôpital Maisonneuve-Rosemont, and Centre hospitalier de l'Université de Montréal). Experience information of the above oncologists was not available. The effective diameter of GTVs has the range of 17–99 mm (mean 40 mm).

### Index images

**Figure 6:**
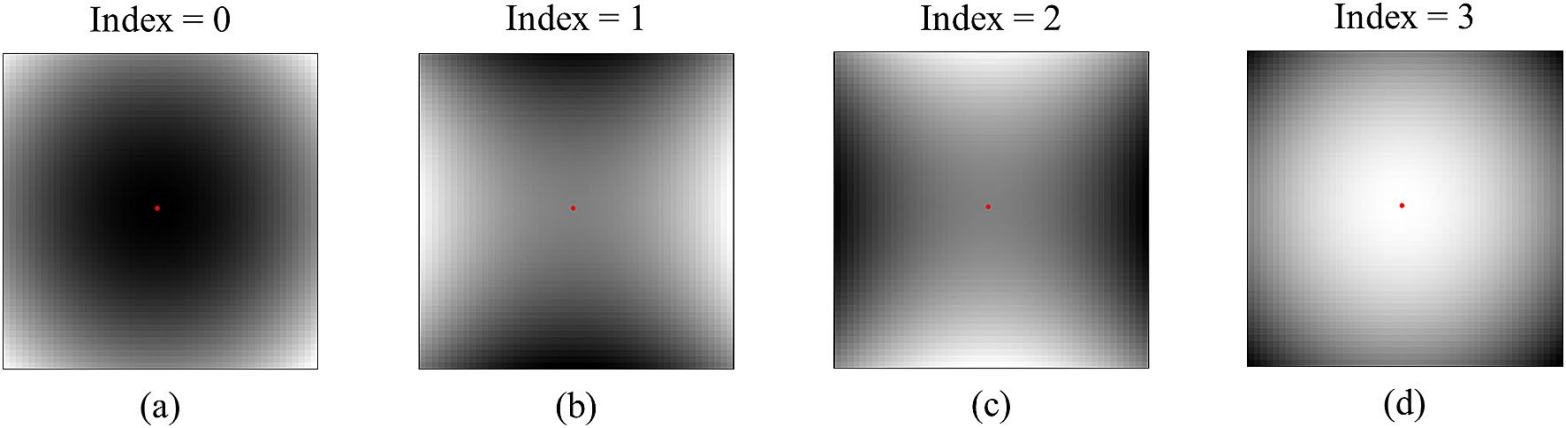
Illustrations of (**a**) concave point with index = 0, (**b**) saddle point with index = 1, (**c**) saddle point with index = 2, and (**d**) convex point with index = 3.

An index image was generated from the Hessian indices at the voxels on a CT image. The Hessian index was calculated as the number of negative eigenvalues of the Hessian matrix^22^ at each voxel on the CT image. The index images could represent the tumor heterogeneity by encoding each voxel with an index: 3 for convex points, 2 or 1 for saddle points, and 0 for concave points. Figure 6 illustrates the cross section of convex, concave, and saddle points on an image.

The partial second derivatives of an image $I(x,y,z)$ first had to be calculated in order to obtain the Hessian matrix of each voxel. However, a 3D Gaussian filter ${G}_{\sigma }\left(x,y,z\right)$ with a standard deviation (SD) $\sigma$ should be convolved with the image to reduce the noise prior to calculating the partial second derivative, because the partial second derivatives are sensitive to image noise^39,40^. In addition to reducing the noise, various scales of tumor heterogeneity could be taken into account by changing the SDs.

The Hessian matrix of each voxel of a 3D CT image $I(x,y,z)$ can be given by

1 $${\varvec{H}}=\left(\begin{array}{ccc}{I}_{xx}\left(x,y,z,\sigma \right)& {I}_{xy}\left(x,y,z,\sigma \right)& {I}_{xz}\left(x,y,z,\sigma \right)\\ {I}_{yx}\left(x,y,z,\sigma \right)& {I}_{yy}\left(x,y,z,\sigma \right)& {I}_{yz}\left(x,y,z,\sigma \right)\\ {I}_{zx}\left(x,y,z,\sigma \right)& {I}_{zy}\left(x,y,z,\sigma \right)& {I}_{zz}\left(x,y,z,\sigma \right)\end{array}\right),$$

where

2 $${I}_{{x}^{i}{y}^{j}{z}^{k}}\left(x,y,z,\sigma \right)=\frac{{\partial }^{2}}{\partial {x}^{i}\partial {y}^{j}\partial {z}^{k}}\left[{G}_{\sigma }\left(x,y,z\right)*I\left(x,y,z\right)\right]=\left[\frac{{\partial }^{2}}{\partial {x}^{i}\partial {y}^{j}\partial {z}^{k}}{G}_{\sigma }\left(x,y,z\right)\right]*I\left(x,y,z\right),$$

$i$, $j$, $k$ are the positive integers satisfying $i+j+k=2$, ${G}_{\sigma }\left(x,y,z\right)$ is the Gaussian filter with a SD $\sigma$, and * denotes convolution.

Let the first, second, and third eigenvalues of ${\varvec{H}}$ be ${\lambda }_{1}$, ${\lambda }_{2},$ and ${\lambda }_{3} \left({\lambda }_{1}\ge {\lambda }_{2}\ge {\lambda }_{3}\right)$, respectively, and the Hessian index (number of negative eigenvalues of ${\varvec{H}}$) be ${i}_{H} \left(0\le {i}_{H}\le 3\right)$. In this study, Jacobi’s method was used for calculating the eigenvalues of the Hessian matrix. The diagonalization of the Hessian matrix is to set a new coordinate system for the matrix. The diagonal form of the Hessian matrix ${\varvec{H}}$ can be expressed as follows:

3 $${\varvec{D}}={{\varvec{U}}}^{T}{\varvec{H}}{\varvec{U}}=\left(\begin{array}{ccc}{\lambda }_{1}& & 0\\ & {\lambda }_{2}& \\ 0& & {\lambda }_{3}\end{array}\right),$$

where ${\varvec{U}}=\left({{\varvec{u}}}_{1}\,{{\varvec{u}}}_{2}\,{{\varvec{u}}}_{3}\right)$ is an orthogonal matrix; ${{\varvec{U}}}^{T}$ is the transposed matrix of ${\varvec{U}}$; and ${{\varvec{u}}}_{1}$, ${{\varvec{u}}}_{2}$, ${{\varvec{u}}}_{3}$ are the eigenvectors of ${\varvec{H}}$. According to Sylvester’s law in linear algebra, the Hessian index is independent on orthogonal matrices ${\varvec{U}}$ or ${{\varvec{U}}}^{T}$, which means that the index is invariant to the choice of coordinate systems.

**Figure 7:**
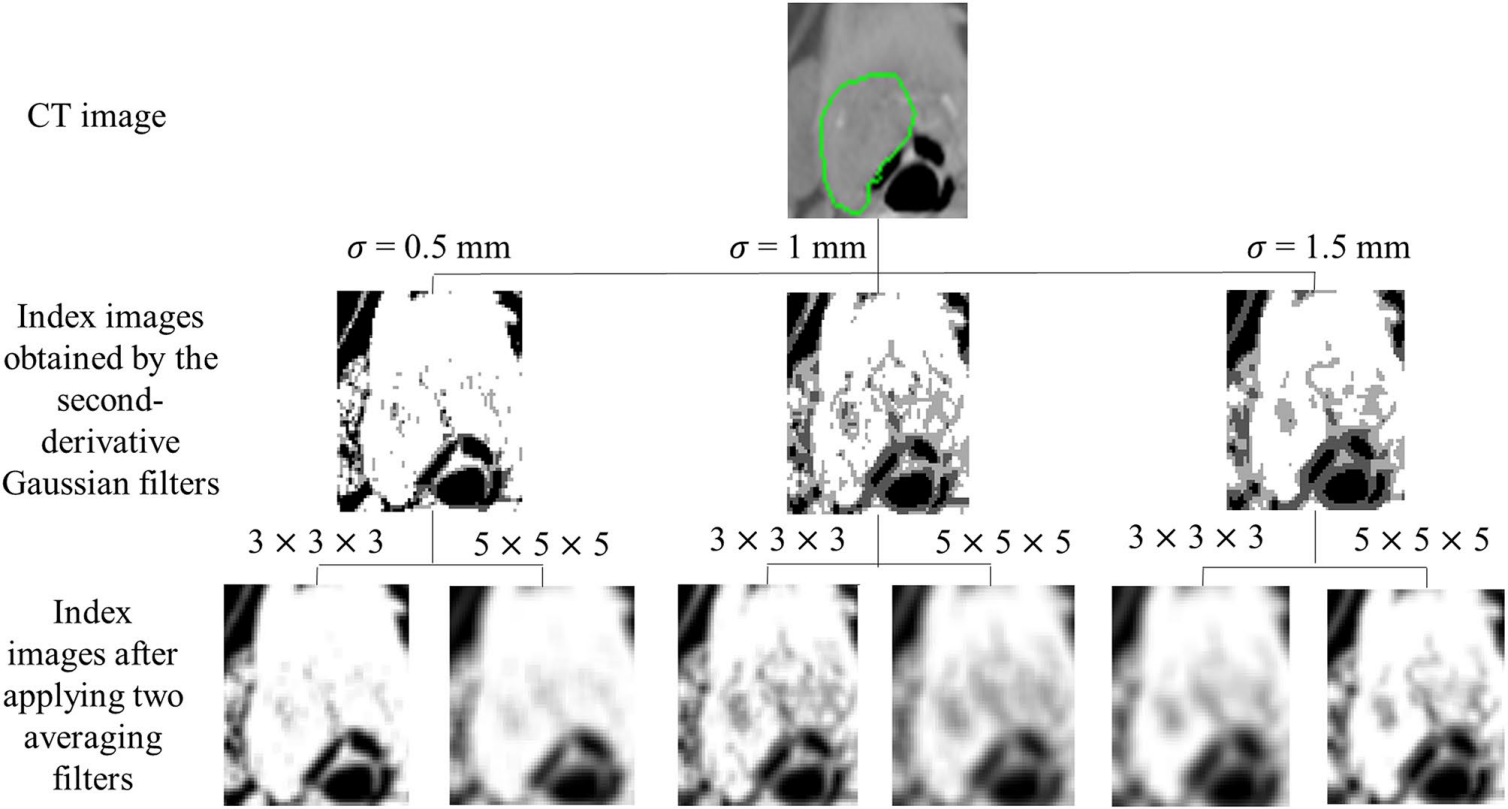
Procedure for generating index images. 1st row: CT image, axial plane; 2nd row: index images obtained by the second-derivative Gaussian filter with SDs of 0.5, 1.0, and 1.5 mm; 3rd row: index images after applying two averaging filters.

Figure 7 illustrates a procedure for generating index images. First, three partial second-derivative Gaussian filters with SDs of 0.5, 1.0, and 1.5 mm, whose corresponding kernel sizes were 3 $\times$ 3 $\times$ 3, 5 $\times$ 5 $\times$ 5, and 7 $\times$ 7 $\times$ 7, respectively, were applied to the original CT images. In image processing, at least three voxels are needed for the calculation of the second derivative based on the first derivative with a half voxel size^50^. Hence, a kernel size of 3 $\times$ 3 $\times$ 3 was used as the smallest kernel. Since the isotropic voxel size was 0.9766 mm, around 1.0 mm may be considered a minimum local heterogeneity in this study. Therefore, three partial second-derivative Gaussian filters with SDs of 0.5, 1.0, and 1.5 mm were employed to take into account multiscale heterogeneity around 1.0 mm in the tumor regions.

Then, two averaging filters with kernel sizes of 3 $\times$ 3 $\times$ 3 and 5 $\times$ 5 $\times$ 5 were convolved with the index images in order to reduce index noise induced by noise present in the CT images, and increase the quantization level of the Hessian index from 2 to 8 bits. As a result, six index images were generated from one CT image, as shown in Fig. 7.

### Calculation of index and conventional features

In this study, radiomic features were categorized into three feature sets: a conventional feature set with features calculated from CT images, an index feature set with features calculated from the index images, and a combined feature set with significant features from the conventional and index feature sets. Conventional and index features were calculated within GTVs from CT and index images, respectively. From the CT images, 486 radiomic features including 14 histogram features, 40 texture features, and 432 wavelet-based features were calculated within the GTVs of each patient. GTVs from CT images were transferred into index images, but only histogram and texture features were calculated. We did not applied wavelet decomposition filters because they might introduce rotational dependency to the index images which are invariant under changing of coordinate systems. Since six index images were generated from one CT image, 324 radiomic features (54 features $\times$ 6 types of images) were calculated from the index images for each patient.

Supplementary Table S1 lists the histogram and texture features used in this study. For the histogram-based features, the CT images were kept with their original Hounsfield values. For the texture features, eight-bit CT images were first acquired based on a look-up table ranging from 0 to 255, which correspond to a range of − 1000 to 1500 Hounsfield units. Texture features were calculated from four texture matrices, that is, a gray level co-occurrence matrix^51^, a gray level run-length matrix^52^, a gray level size-zone matrix^53^, and a neighborhood gray-tone matrix^54^. The texture matrices were constructed by analyzing a volume of interest with a 26 connectivity. Details of the computational procedures are described in the works of Vallières et al.^29^.

In order to extract wavelet-based features from CT images, eight wavelet decomposition filters were applied to decompose the 3D CT images into low and/or high-frequency components at different scales using a coiflet 1 mother wavelet. Eight decomposed images were generated by using eight combinations of low- (scaling function, L) and high-pass filters (wavelet function, H), namely, LLL, HLL, LHL, HHL, LLH, HLH, LHH, and HHH. The procedure for calculating histogram-based and texture features was applied to each wavelet-decomposed image to obtain wavelet-based features. The image preprocessing and computation of wavelet decomposition were performed using an in-house software developed in the Matlab 2018a environment, whereas a Matlab-based Radiomics tools package^29^ was used for the computation of radiomic features.

### Construction of radiomic signature

Radiomic signatures were constructed from signature candidates which were features associated with patients’ prognosis. A Coxnet algorithm, which is similar to the works of Soufi et al.^30^ and Ninomiya and Arimura^31^, was employed to select signature candidates. The regularization term of the Coxnet algorithm was adjusted by changing a blending parameter $\alpha$ to the least absolute shrinkage and selection operator (lasso), which reduced the number of selected features by setting the coefficients of correlated features to exact zero at $\alpha =1$, and the regularization term to ridge regression at $\alpha =0$, thereby selecting more features^30,31,55^.

Instead of preselecting a value of $\alpha$, we optimized $\alpha$ in order to choose the most suitable form of the regularization term for the Coxnet algorithm. The process of optimizing $\alpha$ is detailed in Supplementary Section 2. The Coxnet algorithm was performed in R (R core team, Vienna, Austria) version 3.6.1 using the function *glmnet*^55^ in the package *glmnet*^56^.

For a given feature set, a stepwise forward feature selection using a combination strategy with CPHMs was employed to construct radiomic signatures from signature candidates. The combination strategy used in this study was inspired by the works of Vallières et al.^29^ and is detailed in Supplementary Subsection 3.1.

### Building predictive models using radiomic signatures

For a given feature set, twelve signatures consisting of 1 to 12 features were constructed and twelve corresponding CPHMs were built in the training cohort. We only constructed signatures containing up to twelve features, because the number of features in the signature should not be larger than one tenth of the number of patients in the training cohort^32^ (n = 126). The CPHMs was built in R (R core team, Vienna, Austria) version 3.6.1 using the function *coxph* in the package *survival*.

Streak artifact is a known factor that could affect radiomic models^48^. The impact of streak artifact on the best model built in the training cohort of each feature set was investigated by dividing the test cohort (n = 68) into two groups: an artifact group (n = 20) and a non-artifact group (n = 48). For each feature set, the best model was applied to these two groups to obtain the corresponding log-rank *p* values. The difference between the *p* values in the artifact and non-artifact groups was then calculated. The feature set yielded the lowest difference in *p* value was considered least affected by streak artifact.

### Building predictive models using clinical variables

Clinical variables (i.e. age, T stage, N stage, TNM stage, tumor volume, and HPV status) were also evaluated in term of prediction of prognosis. HPV status was analyzed separately in a univariate analysis because this information was not fully available in our datasets (training cohort: 31/126, test cohort: 13/68). The clinical variables were first converted into numeric values using a conversion table as detailed in Supplementary Table S3. The remaining clinical variables were considered as signature candidates, and the combination strategy was then applied for constructing signature. A CPHM was then built for each signature.

### Building predictive models using radiomic signatures and clinical variables

Signatures from the conventional, index, and combined feature set were integrated with signatures from clinical variables to investigate any potential increments in prognostic power, compared to clinical-variable signatures alone. For a given feature set, because there were five signatures from clinical variables and twelve signatures from radiomic features, sixty integrated signatures were constructed, and a CPHM was built for each integrated signature.

### Evaluation of prognostic powers

The prognostic power of the radiomic models was evaluated based on statistically significant difference (*p* value, log-rank test) between two Kaplan–Meier survival curves that was stratified using the median of the rad-scores. A rad-score is calculated for each patient as a linear combination between each feature in the signature weighted by the corresponding coefficient from the CPHM. The coefficients and median from the training cohort were applied to the test cohort for calculation of rad-scores and stratification of patients, respectively.

## Supplementary information

Supplementary Infomations.

## Data Availability

The combination strategy used in this study is explained in Figure S1 and Algorithm S1 of the Supplementary file. The codes used in this study are available from the corresponding author as long as they will not be related to any patents.
