# Supplementary material for: Radiomic features based on Hessian index for prediction of prognosis in head-and-neck cancer patients
Source: Sci Rep. 2020 Dec 4;10:21301. doi: 10.1038/s41598-020-78338-7 (PMC7718925; doi:10.1038/s41598-020-78338-7)
Supplement: Supplementary file 1 — Supplementary Infomations. [file 41598_2020_78338_MOESM1_ESM.docx]

**RADIOMIC FEATURES BASED ON HESSIAN INDEX FOR PREDICTION OF PROGNOSIS IN HEAD-AND-NECK CANCER PATIENTS**

**Quoc Cuong Le^1^, Hidetaka Arimura^2*^, Kenta Ninomiya^1^ and Yutaro Kabata^3^**

^1^ Department of Health Sciences, Graduate school of Medical Sciences, Kyushu University, Japan

^2*^ Department of Health Sciences, Faculty of Medical Sciences, Kyushu University, Japan

^3^ Institute of Mathematics for Industry, Kyushu University, Japan

Email: arimurah@med.kyushu-u.ac.jp

**1. Calculation of radiomic features**

Fourteen histogram-based and 40 texture features used in this study are listed in table S1.

|  | Texture type | Reference(s) | Feature name |
| --- | --- | --- | --- |
| Histogram-based features | - | - | Energy  Entropy  Kurtosis  Maximum  Mean  Mean absolute difference (MAD)  Median  Minimum  Range  Root mean square (RMS)  Skewness  Standard deviation (STD)  Uniformity  Variance |
| Texture features | Gray-level co-occurrence matrix (GLCM) | (Haralick *et al.*^1^) | Energy  Contrast  Entropy  Homogeneity  Correlation |
|  |  | (Haralick *et al*.^1^, Assefa *et al.*^2^) | Sum Average  Variance |
|  |  | (Thibault^3^) | Dissimilarity |
|  |  | (Aerts *et al.*^4^) | Auto correlation |
|  | Gray-level run-length matrix (GLRLM) | (Galloway^5^) | Short run emphasis (SRE)  Long run emphasis (LRE)  Gray-level nonuniformity (GLN)  Run-length nonuniformity (RLN)  Run percentage (RP) |
|  |  | (Chu *et al.*^6^) | Low gray-level run emphasis (LGRE)  High gray-level run emphasis (HGRE) |
|  |  | (Dasarathy and Holder^7^) | Short run low gray-level emphasis (SRLGE)  Short run high gray-level emphasis (SRHGE)  Long run low gray-level emphasis (LRLGE)  Long run high gray-level emphasis (LRHGE) |
|  |  | (Thibault *et al.*^8^) | Gray-level variance (GLV)  Run-length variance (RLV) |
|  | Gray-level size zone matrix (GLSZM) | (Galloway^5^, Thibault *et al*.^8^) | Small zone emphasis (SZE)  Large zone emphasis (LZE)  Gray-level nonuniformity (GLN)  Zone-size nonuniformity (ZSN)  Zone percentage (ZP) |
|  |  | (Chu *et al.*^6^, Thibault *et al.*^8^) | Low gray-level zone emphasis (LGZE)  High gray-level zone emphasis (HGZE) |
|  |  | (Dasarathy and Holder^7^, Thibault *et al.*^8^) | Small zone low gray-level emphasis (SZLGE)  Small zone high gray-level emphasis (SZHGE)  Large zone low gray-level emphasis (LZLGE)  Large zone high gray-level emphasis (LZHGE) |
|  |  | (Thibault *et al.*^8^) | Gray-level variance (GLV)  Zone-size variance (ZSV) |
|  | Neighborhood gray-tone difference matrix (NGTDM) | (Amadasun and King^9^) | Coarseness  Contrast  Busyness  Complexity  Strength |

**Table S1.** Histogram-based and texture features used in this study.

**2. Selection of signature candidates**

In order to select signature candidates, a Coxnet algorithm based on a multivariate Cox proportional hazards regression model^10^ (CPHM) regularized with an elastic net^11-13^ was employed. The blending parameter$\alpha$ was optimized based on a leave-one-out cross validation (LOOCV) by changing it from 0.01 to 1 (step 0.01) for the training cohort, and minimizing the mean cross-validation error in LOOCV for 100 iterations in the Coxnet algorithm.

**3. Construction of radiomic signatures**

*3.1. Stepwise forward feature selection with combination strategy*

The combination strategy with CPHMs was inspired by the works of Vallières *et al.*^14^. Figure S1 illustrates the workflow of the combination strategy, in which a model of order N (the number of features in the combination used to construct that model) was constructed. Algorithm S1 shows the pseudo code of the combination strategy. In the combination strategy, the training cohort was randomly divided into a training set $\left( S_{\mathrm{train}} \right)$ constituted by 64 patients and a validation set $\left( S_{\mathrm{val}} \right)$ constituted by 28 patients. The functions for generating uniform random number (runif) and specify the state of random number generation (set.seed) in R (R core team, Vienna, Austria) version 3.6.1 were used for the random division. The purpose of the division was to select a combination that could produce a robust predictive model to unknown data.

For a given feature set, multiple combinations containing 1-N features were formed and a CPHM was constructed for each combination in the set$S_{\mathrm{train}}$. The CPHM was internally evaluated in the set $S_{\mathrm{train}}$ based on a Kaplan-Meier analysis to obtain a log-rank p-value ($\mathrm{pval}_{S_{\mathrm{train}}}$), and externally evaluated in the set $S_{\mathrm{val}}$ to obtain a log-rank p-value ($\mathrm{pval}_{S_{\mathrm{val}}}$). For selecting a combination which could produce a robust model to unknown data (e.g. balance between $\mathrm{pval}_{S_{\mathrm{train}}}$and $\mathrm{pval}_{S_{\mathrm{val}}}$), the following cost function was optimized:

| $F=\frac{L_{T}+L_{V}}{1+\left\vert L_{T}-L_{V} \right\vert},$ | (1) |
| --- | --- |

where $L_{T}$ is $-\log_{10} \left( \mathrm{pval}_{S_{\mathrm{train}}} \right)$, and $L_{V}$ is $-\log_{10} \left( \mathrm{pval}_{S_{\mathrm{val}}} \right)$.

In this study, among multiple CPHMs with the same order, a model yielding the highest value of the cost function $F$ was constructed, and the combination, which produced the model, was determined.

| 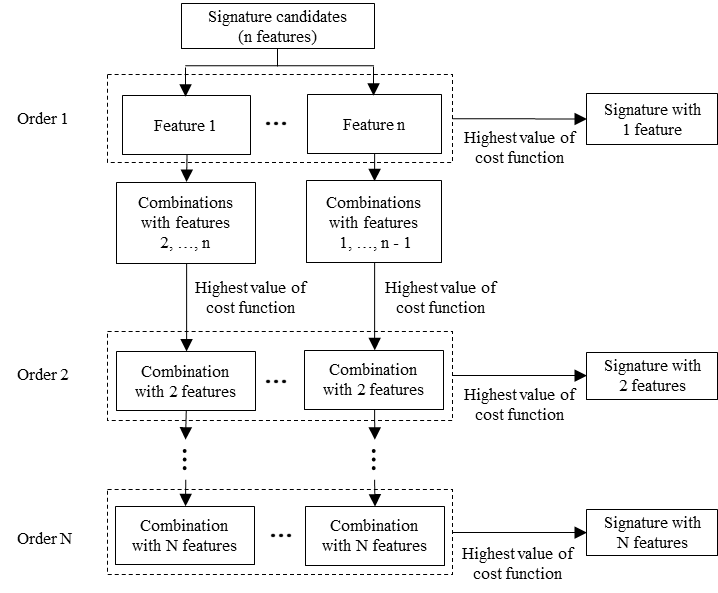 |
| --- |
| **Figure S1.** A workflow of the combination strategy. |

| **Algorithm S1**: A pseudo code of a combination strategy  **Input**: signatures candidates, desired model order N, train set $S_{\mathrm{train}}$, validation set $S_{\mathrm{val}}$  **Output**: signatures containing 1 to N features |
| --- |
| *maxModelOrder* = *N*  **for** *i* = 1 to number of signature candidates **do**  *starter* = feature *i*  **for** *modelOrder* = 1 to *maxModelOrder* **do**  **if** *modelOrder* == 1  *combination* = *starter*  Construct a CPHM using *combination* in set $S_{\mathrm{train}}$ to obtain $\mathrm{pval}_{S_{\mathrm{train}}}$  Test the CPHM in set $S_{\mathrm{val}}$ to obtain $\mathrm{pval}_{S_{\mathrm{val}}}$  Calculate cost function $F$ according to equation (1)  Select the combination  **elseif** *modelOrder* > 1  **for** *j* = 1 to number of signature candidates and *j* $\neq$ *i* **do**  *combination* = *starter* and feature *j*  Construct an CPHM using *combination* in set $S_{\mathrm{train}}$ to obtain $\mathrm{pval}_{S_{\mathrm{train}}}$  Test the CPHM in set $S_{\mathrm{val}}$ to obtain $\mathrm{pval}_{S_{\mathrm{val}}}$  Calculate cost function $F$ according to equation (1)  **endfor**  Identify the model yielded the highest value of cost function $F$ and select its corresponding *combination*  **endif**  *starter* = *combination*  **endfor**  **endfor**  **for** *i* = 1 to *maxModelOrder* **do**  Among models of order *i*, select the model having the highest cost function $F$ and its corresponding *combination*  Use the selected *combination* as a signature with *i* features  **endfor** |

For a given feature set and CPHM, the selection step was divided into multiple experiments, and the number of experiments depended on the number of signature candidates in the given feature set. In each experiment, a different signature candidate was used as a ‘starter’. For a given *starter*, a CPHM of order 1 was constructed in the set $S_{\mathrm{train}}$. The constructed model was internally and externally evaluated in sets $S_{\mathrm{train}}$ and$S_{\mathrm{val}}$, respectively. Two values of log-rank p-values (i.e. $\mathrm{pval}_{S_{\mathrm{train}}}$ and$\mathrm{pval}_{S_{\mathrm{val}}}$) were then obtained, and a value of the cost function (1) was calculated. Since there was only one CPHM of order 1 constructed using the given *starter*, the given *starter* was recorded without further consideration. Next, multiple combinations of two features consisting of the *starter* and each of the remaining signature candidates were formed, and a CPHM of the same order was constructed for each combination in the set $S_{\mathrm{train}}$. Each model was internally and externally evaluated in sets $S_{\mathrm{train}}$ and $S_{\mathrm{val}}$ to obtain $\mathrm{pval}_{S_{\mathrm{train}}}$ and$\mathrm{pval}_{S_{\mathrm{val}}}$, respectively, and a value of the cost function was calculated. Then, the model yielded the highest value of the cost function was selected and the combination used to construct that model was recorded. This process was repeated up to model order N, i.e. a model constructed using a signature with N features.

After finishing all experiments, the number of models with the same order were equal to the number of experiments. For a given model order, the experiment maximizing the cost function was identified, and the combination corresponding to that model order was considered a signature. As a result, N signatures were selected, one for each model order. It should be noticed that CPHMs constructed so far are just for the purpose of constructing signatures.

*3.2. Best signature of each feature set*

Table S2 lists features in the best signature and integrated signature for each feature set.

| Signature | | |
| --- | --- | --- |
| Conventional feature set | Index feature set | Combined feature set |
| HHH_Hist_Skew | k5s0.5_Hist_MAD | GLSZM_LZE |
| Hist_Mean | k5s1.5_NGTDM_Busyness | k3s0.5_GLCM_Variance |
| Hist_Skew |  | NGTDM_Contrast |
| HLL_Hist_Skew |  | Hist_Skew |
| HHL_Hist_Median |  | HHH_Hist_Skew |
| HHL_Hist_Skew |  | HHH_GLSZM_LZE |
| LLH_GLSZM_LZHGE |  | HHH_GLSZM_GLV |
| LLL_Hist_Skew |  |  |
| GLSZM_GLV |  |  |
| Integrated signature | | |
| Conventional + clinical | Index + clinical | Combined + clinical |
| LLH_GLSZM_LZHGE | k3s1_GLSZM_LZHGE | GLSZM_LZE |
| LLL_GLSZM_ZSN | k5s0.5_Hist_Variance | k3s0.5_GLCM_Variance |
| HHH_Hist_Skew | k5s0.5_GLSZM_SZE | NGTDM_Contrast |
| HLH_Hist_Median | k5s1_Hist_STD | Hist_Skew |
| LHL_Hist_Mean | k5s0.5_GLRLM_SRLGE | HHH_Hist_Skew |
| Age | k3s1_NGTDM_ Busyness | HHH_GLSZM_LZE |
| T stage | k3s1.5_GLRLM_SRHGE | HHH_GLSZM_GLV |
| TNM stage | k5s1_GLSZM_GLV | Age |
| N stage | k5s1_Hist_MAD | T |
| Volume | Age | TNM stage |
|  | T stage | N |
|  | TNM stage | Volume |
|  | N stage |  |
|  | Volume |  |

**Table S2.** Best signature and integrated signature for each feature set.

In the above signatures, features whose name starts with the combination of the letter k and s are index features. The letter k indicates the kernel size of the averaging filter (i.e. k3 and k5 imply 3$\times$3$\times$3 and 5$\times$5$\times$5 filters, respectively) and s indicates the standard deviation (SD) of the second-derivative Gaussian filter (i.e. s0.5, s1, and s1.5 imply Gaussian filters whose SDs are 0.5, 1, and 1.5 mm, respectively). Hence, the combination of k and s indicates the type of index image from which the radiomic feature was calculated (e.g. k3s0.5).

**4. *Conversion of character values of clinical data***

The clinical variables were first converted into numeric values using a conversion table prior to constructing CPHMs. Tables S3 illustrates the conversion table used in this study.

| T stage | | N stage | | TNM stage | | HPV | |
| --- | --- | --- | --- | --- | --- | --- | --- |
| Character value | Numeric value | Character value | Numeric value | Character value | Numeric value | Character value | Numeric value |
| T0 | 1 | N0 | 1 | I | 1 | NA | Exclude |
| T1 | 2 | N1 | 2 | II | 2 | Negative | 1 |
| T2 | 3 | N2 | 3 | III | 3 | Positive | 2 |
| T3 | 4 | N2a | 4 | IV | 4 |  |  |
| T3a | 5 | N2b | 5 | IVA | 5 |  |  |
| T4 | 6 | N2c | 6 | IVB | 6 |  |  |
| T4a | 7 | N3 | 7 |  |  |  |  |
| T4b | 8 | N3a | 8 |  |  |  |  |
|  |  | N3b | 9 |  |  |  |  |

**Table S3.** Conversion table for clinical data.

**References**

1. Haralick, R. M., Shanmugam, K. & Dinstein, I. Textural features for image classification *IEEE Trans. Syst. Man Cybern.* **3**, 610-21 (1973).

2. Assefa, D., Keller, H., Ménard, C., Laperriere, N., Ferrari, J. R. & Yeung, I. Robust texture features for response monitoring of glioblastoma multiforme on T1-weighted and T2-FLAIR MR images: A preliminary investigation in terms of identification and segmentation *Med. Phys.* **34(4)**, 1722-36 (2010).

3. Thibault, G. Indices de formes et de textures: de la 2D vers la 3D. Application au classement de noyaux de cellules *PhD Thesis* University AIX-Marseille, France (2009).

4. Aerts, H. J. W. L. *et al* Decoding tumour phenotype by noninvasive imaging using a quantitative radiomics approach *Nat. Commun.* **5**, 4006 (2014).

5. Galloway, M. M. Texture analysis using gray level run lengths *Comput. Graph. Image Process.* **4**, 172-9 (1975).

6. Chu, A., Sehgal, C. & Greenleaf, J. Use of gray level distribution of run lengths for texture analysis *Pattern Recognit. Lett.* **11**, 415-9 (1990).

7. Dasarathy, B. & Holder, E. Image characterizations based on joint gray-level run length distributions *Pattern Recogn. Lett.* **12**, 497-502 (1991).

8. Thibault, G., Fertil, B., Navarro, C., Pereira, S., Cau, P., Levy, N., Sequeira, J. & Mari, J. L. Texture indexes and gray level size zone matrix: application to cell nuclei classification *Pattern Recognition and Information Processing (PRIP) (Minsk, Belarus)* pp 140–5 (2009).

9. Amadasun, M. & King, R. Textural features corresponding to textural properties IEEE *Trans. Syst. Man Cybern.* **19**, 1264–74 (1989).

10. Cox, D. R. Regression models and life tables (with discussion) *J. R. Statist. Soc. B* **34**, 187-220 (1972).

11. Simon, N., Friedman, J., Hastie, T. & Tibshirani, R. Regularization paths for Cox’s proportional hazards model via coordinate descent *J. Stat. Soft.* **39(5)**, 1-13 (2011).

12. Soufi, M., Arimura, H. & Nagami, N. Identification of optimal mother wavelets in survival prediction of lung cancer patients using wavelet decomposition-based radiomic features *Med. Phys.* **45**, 5116–28 (2018).

13. Ninomiya, K. & Arimura, H. Homological radiomics analysis for prognostic prediction in lung cancer patients *Phys. Medica* **69**, 90-100 (2020).

14. Vallières, M., Freeman, C. R., Skamene, S. R. & El Naqa, I. A radiomics model from joint FDG-PET and MRI texture features for the prediction of lung metastases in soft-tissue sarcomas of the extremities *Phys. Med. Biol.* **60**, 5471-5496 (2015).
